# Draft Genome Sequence of a Hospital-Associated Clone of *Klebsiella pneumoniae* ST340/CC258 Coproducing RmtG and KPC-2 Isolated from a Pediatric Patient

**DOI:** 10.1128/genomeA.01130-16

**Published:** 2016-11-03

**Authors:** Louise Cerdeira, Miriam R. Fernandes, Gabriela R. Francisco, Maria Fernanda C. Bueno, Susan Ienne, Tiago A. Souza, Doroti de Oliveira Garcia, Nilton Lincopan

**Affiliations:** a Department of Clinical Analysis, School of Pharmacy, Universidade de São Paulo, São Paulo, Brazil; b Center of Bacteriology, Instituto Adolfo Lutz, São Paulo, Brazil; c Genome Investigation and Analysis Laboratory (GENIAL), Institute of Biomedical Sciences, Universidade de São Paulo, São Paulo, Brazil; d Department of Microbiology, Institute of Biomedical Sciences, Universidade de São Paulo, São Paulo, Brazil

## Abstract

We report here the draft genome sequence of a *Klebsiella pneumoniae* strain 1194/11, belonging to the hospital-associated sequence type 340 (ST340; clonal complex CC258), isolated from a catheter tip culture from a pediatric patient. The multidrug-resistant strain coproduced the 16S rRNA methyltransferase rRNA RmtG and β-lactamases KPC-2 and CTX-M-15.

## GENOME ANNOUNCEMENT

*Klebsiella pneumoniae* is a leading cause of nosocomial infection. In this regard, clinical strains exhibiting a multidrug-resistant (MDR) profile have been associated with the persistence of clonally related sequence types (STs), clustered in high-risk complexes (1). In this regard, most KPC-2-producing *K. pneumoniae* strains belong to the clonal complex 258 (CC258), which includes ST11, ST258, ST340, and ST437 (1). In Brazil, strains of *K. pneumoniae* ST340 coproducing KPC-2 and CTX-M-15 have been found in clinical settings and aquatic ecosystems (2–4). Here, we present the draft genome sequence of an MDR ST340 *K. pneumoniae* strain coproducing the 16S rRNA methyltransferase rRNA RmtG and β-lactamases KPC-2 and CTX-M-15.

*K. pneumoniae* strain 1194/11 was isolated in 2011 from a catheter tip culture from a pediatric patient admitted to a children’s hospital in São Paulo, southern Brazil (5). The strain was collected at the Instituto Adolfo Lutz (IAL), which serves as a state reference laboratory and receives multidrug-resistant Gram-negative pathogens on an ongoing basis. The isolate belonged to ST340/CC258 and displayed resistance to gentamicin, tobramycin, amikacin, arbekacin, neomycin, apramycin, ceftazidime, cefotaxime, cefepime, ertapenem, and meropenem (5).

The genomic DNA of *K. pneumoniae* strain 1194/11 was sequenced using an Illumina MiSeq sequencing platform, which produced 1,824,432 paired-end reads at 100× total coverage. *De novo* assembly was performed using the A5-MiSeq pipeline (6), and the assembled contigs were annotated using NCBI Prokaryotic Genome Annotation Pipeline version 3.2. In total, there were 5,643 protein-coding genes, 123 RNA-coding genes (81 tRNAs, 27 rRNAs, and 15 noncoding RNAs [ncRNAs]), and 108 pseudogenes, with a G+C content of 56.7% and estimated 5,702,010-bp genome size.

The resistome of *K. pneumoniae* strain 1194/11 was analyzed using ResFinder 2.1 (7), identifying *aac(3)-IId*, *rmtG*, and *aadA2* aminoglycoside resistance genes; *bla*_CTX-M-15_, *bla*_SHV-11,_ *bla*_KPC-2_, and *bla*_TEM-1B_ β-lactam resistance genes; the *sul1* sulfonamide resistance gene; the *tet*(D) gene (conferring resistance to tetracycline); trimethoprim resistance genes *dfrA12* and *dfrA25*; the fosfomycin resistance *fosA*-like gene, and plasmid-mediated quinolone resistance (PMQR) genes *oqxA*, *oqxB*, and *qnrB2*.

### Accession number(s).

The genome sequence of *Klebsiella pneumoniae* 1194/11 has been deposited in the GenBank database under the accession number LYZC00000000.
